# A Comparison of Novel Serum Markers of Liver Health in Adolescents With Metabolic Dysfunction‐Associated Steatotic Liver Disease

**DOI:** 10.1111/jcmm.70817

**Published:** 2025-08-28

**Authors:** Neeharika Bade, Diana A. Hellman, David J. Matye, Shaoning Jiang, Jeanie B. Tryggestad, Zhongxin Yu, Kevin R. Short, Sirish K. Palle

**Affiliations:** ^1^ Section of Gastroenterology, Hepatology, and Nutrition University of Oklahoma Health Sciences Center Oklahoma City Oklahoma USA; ^2^ Section of Diabetes and Endocrinology, Department of Pediatrics University of Oklahoma Health Sciences Center Oklahoma City Oklahoma USA; ^3^ Department of Pharmacology, Toxicology and Therapeutics University of Kansas Medical Center Kansas City Kansas USA; ^4^ Department of Pathology University of Oklahoma Health Sciences Center Oklahoma City Oklahoma USA

**Keywords:** fibrosis, MASLD, microRNA, obesity, paediatric

## Abstract

Several non‐invasive biomarkers for paediatric metabolic dysfunction‐associated steatotic liver disease (MASLD) have been reported, but no prior studies directly compared multiple protein or microRNA (miRNA) markers of liver health in adolescents with and without MASLD and determined which serum markers are associated with liver histopathological features. We measured 6 serum protein and 4 miRNA candidates in 3 groups of participants: 23 with obesity and biopsy‐proven MASLD, 24 controls with obesity (Ob) and 24 controls with normal weight (NW). The MASLD group had higher median values for cytokeratin 18 (CK‐18, 8.5 and 5.6‐fold higher than NW and Ob, respectively), CK‐18 fragments (2.6‐ and 2.6‐fold), collagen IV (0.9‐ and 0.6‐fold), miR‐122 (16.9‐ and 10.7‐fold) and miR‐192 (9.7‐ and 12.0‐fold). YKL‐40 and N‐terminal propeptide of type III procollagen were only higher in the MASLD group compared to the NW group. Serum AST, CK‐18, CK‐18 fragments, miR‐122 and miR‐192 were positively correlated with liver fibrosis stage. Area under the receiver operating curve for identifying MASLD for CK‐18 (0.962), miR‐192 (0.945) and miR‐122 (0.944) was higher than ALT (0.935). miR‐122 in serum and liver was inversely correlated in MASLD patients but neither was associated with putative mRNA targets *AGPAT1* and *DGAT1*. These results show that CK‐18, miR‐122 and miR‐192 are marginally better predictors of MASLD than ALT and correlated with fibrosis in this cohort, supporting further work to confirm these findings.

**Abbreviations**
AGPAT1: 1‐acyl‐sn‐glycerol‐3‐phosphate acyltransferase alpha
ALT: alanine amino transferase
AST: aspartate aminotransferase
BMI: body mass index
CK‐18: cytokeratin 18
Col‐IV: collagen type IV
DGAT1: diglyceride acyltransferase 1
HbA1c: glycated haemoglobin
iHOMA2: interactive homeostatic model of assessment
MASH: metabolic dysfunction‐associated steatohepatitis
MASLD: metabolic disease‐associated steatotic liver disease
miRNA: microRNA
NEFA: non‐esterified fatty acids
NW: normal weight
Ob: obesity
PIIINP: N‐terminal pro‐collagen III propeptide
SREBP1: sterol regulatory element‐binding protein 1
VO_2_peak: peak rate of oxygen uptake
YKL‐40: chitinase‐3‐like protein 1

## 1 Introduction

Metabolic dysfunction‐associated steatotic liver disease (MASLD) is the leading chronic liver disease among adolescents, driven largely by the global rise in obesity [1, 2]. MASLD encompasses a spectrum of progressive liver pathologies, ranging from simple steatosis to steatohepatitis (MASH), advanced fibrosis and cirrhosis [3]. A challenge in MASLD management is the lack of reliable, minimally invasive biomarkers capable of identifying disease severity and monitoring progression or therapeutic response over time [4]. The best currently available blood biomarker for MASLD is alanine aminotransferase (ALT) [3]. While ALT is useful for identifying potential MASLD, it lacks specificity and does not reliably indicate disease severity or the progression to MASH [5]. Better biomarkers for MASLD could reduce the need for liver biopsies, the current gold‐standard approach for MASLD confirmation, especially during long‐term follow‐up [6, 7]. Although some features of MASLD are similar in adults and children, paediatric MASLD progression differs from that in adults, underscoring the need for targeted research to determine the most reliable biomarkers in young people [8].

Chronic liver steatosis and inflammation result in apoptosis and necrosis, induce repair and remodelling responses, and release several potential biomarkers into the circulation. These include commonly measured liver enzymes (e.g., ALT and aspartate aminotransferase, AST), proteins like chitinase‐3‐like protein 1 (YKL‐40), and constituents of the extracellular matrix like hyaluronic acid, collagen type IV (Col‐IV), N‐terminal pro‐collagen III propeptide (PIIINP), cytokeratin 18 (CK‐18) and CK‐18 fragments [7, 9, 10, 11, 12, 13]. PIIINP, CK‐18 and CK‐18 fragments were reported to be correlated with liver injury and/or fibrosis in children [10, 11, 12, 13]. YKL‐40 and Col‐IV have shown potential in differentiating between stages of fibrosis in adults with MASLD, but have not yet been reported in adolescents [9, 14].

Circulating micro‐RNAs (miRNA) may also be useful for MASLD detection. miRNAs are small, non‐coding RNAs that regulate protein expression by binding specific mRNAs [15, 16]. Serum and liver content of several miRNAs are altered in adults and animal models of MASLD [15, 17, 18, 19], but there are far fewer investigations of miRNAs in paediatric MASLD [20, 21, 22]. miR‐122 is a promising MASLD biomarker because it is predominantly produced in the liver, is abundant in the liver and blood, and its serum content increases with the severity of MASLD in adults and animals [16, 19, 23, 24, 25]. The transcriptional targets of miR‐122 include the triglyceride‐synthesis enzymes 1‐acyl‐sn‐glycerol‐3‐phosphate acyltransferase alpha (*AGPAT1*) and diglyceride acyltransferase (*DGAT1*), which are suppressed in healthy livers of rodents and hepatocyte cultures when fatty acid concentrations rise [16, 26]. During the progression of simple steatosis to MASH, miR‐122 is increasingly exported to the blood, presumably reducing its inhibitory effect on liver lipogenesis [16, 25]. The first study that compared serum miR‐122 in children with MASLD to a control group reported that miR‐122 was elevated in one cohort of children with the disease, but unaffected in another, with no clear explanation for that discrepancy [20]. Another study reported that serum miR‐122, ‐192 and 34a were increased in children and adolescents with MASLD compared to peers with obesity but included participants with type 2 diabetes, which could potentially confound the effect of MASLD alone [22]. The only study of miRNAs associated with MASLD in adolescents that included liver tissue analyses, performed in patients undergoing bariatric surgery, reported that serum miR‐122 was positively associated with the severity of liver fibrosis [21]. miR‐192 is also abundant in liver and serum and has promise as a predictor of liver histopathology in adults but has not been reported in paediatric patients [19, 24, 27, 28]. Another miRNA with a role in MASLD is miR‐155, which represses transcripts for fatty acid synthase and one of its regulators, sterol regulatory element‐binding protein 1 (*SREBP1*) in hepatocytes, leading to lower triglyceride production [29]. Adults with MASLD had reduced serum and liver miR‐155 content compared to a control group, while liver transcripts for *SREBP1* and fatty acid synthase were increased [29]. miR‐155 promotes adipose macrophage activation, liver inflammation and insulin resistance [30, 31] but has not been reported in paediatric patients with MASLD.

While these biomarkers hold promise, their assessment in paediatric populations is incomplete. No prior studies directly compared multiple protein or miRNA markers of liver health in paediatric patients with and without MASLD and determined which serum markers are associated with liver histopathological features. To address this gap, we measured six proteins and four miRNAs in serum as putative biomarkers of MASLD in adolescents with biopsy‐confirmed MASLD and control groups with normal weight (NW) or obesity (Ob). Within the MASLD group, we also measured the same miRNAs in liver biopsies, along with transcripts for five key proteins involved in lipogenesis and its regulation. We tested whether liver miRNAs and mRNAs were associated with one another or liver histopathology.

## 2 Methods

### 2.1 Protocol and Ethical Approval

This was a cross‐sectional pilot study performed at a single location and as such, a power calculation was not determined a priori. Participants were enrolled between November 2018 and August 2020. Informed written consent and assent of all participants (and their parent or guardian as appropriate) were obtained in accordance with the guidelines of the University of Oklahoma Health Sciences Institutional Review Board, which approved the study.

### 2.2 Participants

Boys and girls 12 to 20 years old were recruited into one of three categories: (1) Controls with NW and without MASLD, (2) Controls with Ob and without MASLD, or (3) patients with Ob and biopsy‐proven MASLD. Normal weight was defined as having a body mass index (BMI) between the 5th and 84th percentile using Centers for Disease Control growth charts [32], while obesity was defined as ≥ 95th percentile for both the Ob and MASLD groups. There was not an upper limit for BMI. Participants in the MASLD group were recruited from the MASLD clinic when they were scheduled for liver biopsy as part of their routine care. NW and Ob controls were recruited from the community. The Ob and NW control groups were selected to have similar age, sex and race/ethnicity distribution as the MASLD group, but with no clinical evidence of liver disease, including a serum ALT value < 47 U/L (the healthy reference range for our laboratory analyser), upper abdominal pain, severe fatigue, or unexplained weight loss or weakness. Inclusion criteria for the MASLD group were persistently elevated serum ALT, ultrasound findings consistent with MASLD, and biopsy findings confirming MASLD. Patients with steatosis due to factors such as medications, viral hepatitis, genetic or metabolic conditions, alcohol consumption, drug abuse, or a history of total parenteral nutrition were excluded from the study by the paediatric hepatologist who ran the MASLD clinic. Participants with diabetes were also excluded from all groups. The absence of diabetes was confirmed by measuring glycated haemoglobin (HbA1c, which had to be < 6.5%), fasting blood glucose (which had to be < 7.0 mmol/L), thorough medical history, and review of medical records (when available). We expected that the patients in the MASLD group would have low habitual physical activity so to reduce the potential confounding effects of exercise on metabolic outcomes, candidates for the NW and Ob control groups were excluded if they were engaged in structured exercise programs on more than 3 days per week for the previous 3 months. A paediatric endocrinologist performed a basic exam and history to confirm that all participants had entered puberty (defined as Tanner stage 2 or greater, based on development of breasts (girls) or pubic hair (boys)). Full Tanner staging was not performed to minimise the invasiveness of the screening visit. Pre‐pubertal children were excluded to limit potential effects of changes in hormones, growth factors and insulin resistance on the measured variables.

### 2.3 Physiological Tests

Laboratory‐based blood collection and physiological tests were performed in the morning following an overnight fast. Participants were instructed to avoid vigorous physical activity and maintain their habitual diet on the day prior to testing, and to stop eating food and caffeinated beverages for ten hours prior to the start of the visit.

Height and weight were measured on a calibrated stadiometer and scale, respectively. Those values were used to calculate BMI (kg/m^2^, percentile and *z*‐score) [32]. Participants in the Ob and MASLD groups were classified as having Class 1, 2, or 3 obesity according to the following definitions: Class 1: BMI > 95th percentile and < 120% of the 95th percentile; Class 2: BMI ≥ 120% and < 140% of the 95th percentile; Class 3: BMI > 40 kg/m^2^ or > 140% of the 95th percentile [32]. Body composition was measured using dual energy X‐ray absorptiometry (GE/Lunar iDXA, GE‐Healthcare, Fairfield, CT). A total body scan was performed and values for total lean soft tissue mass, total fat mass (absolute and as a percentage of body mass) and trunk fat (absolute and as a percentage of trunk mass) are reported. Aerobic fitness was measured as the peak rate of oxygen consumption (VO_2_ peak) during a graded exercise test on a stationary bicycle. Daily ambulatory activity was measured with an accelerometer (StepWatch 3, OrthoCare Innovations, Oklahoma City, OK) worn above the ankle during waking hours, recording data every minute for seven days. Data were used for analysis if there was at least 10 h of recorded activity on at least 5 days.

### 2.4 Blood Analyses

Venous blood was collected while fasting. Glycated haemoglobin (HbA1c) was measured on whole blood using a Siemens DCA Vantage analyser (Tarrytown, NY). The remaining blood was centrifuged within 30 min of collection, and aliquots of plasma and serum were stored at −80°C until analysis. Plasma glucose was measured with the glucose oxidase method (Fujifilm Wako Diagnostics USA Corp., Mountainview, CA). Serum insulin was measured using an ELISA (#80‐INSHU‐CH01, Alpco, Salem, NH). Insulin resistance was calculated using glucose and insulin concentrations with the revised integrated homeostatic model of assessment (iHOMA2) [33]. Serum total cholesterol, HDL‐cholesterol, triglycerides, ALT and AST were measured using a Piccolo Xpress clinical chemistry analyser (Piccolo, Abbott, Princeton, NJ). LDL‐cholesterol was calculated [34]. Serum non‐esterified fatty acids (NEFA) were measured using a colorimetric assay (Fujifilm Wako). Protein biomarkers in serum were measured with commercial ELISAs. Full‐length CK‐18 (M65) and CK‐18 fragments (M30) assays were from Abcam (#ab227896 and #ab254515, respectively, Waltham, MA). PIIINP and Col‐IV assays were from Novus Biologicals, (#NBP2‐76434 and #NBP2‐75864, respectively, Centennial, CO). Hyaluronic acid and C‐reactive protein assays were from R&D Systems (#DHYAL0 and #DCRP00B, respectively, Minneapolis, MN). The YKL‐40 assay was from Thermo Fisher (#BMS2322, Waltham, MA). All samples were analysed in duplicate according to the manufacturer's instructions.

Serum abundance of selected miRNAs was measured after isolating total RNA from equal volumes of serum with the miRNeasy Serum/Plasma Advanced Kit from Qiagen (Germantown, MD). Reverse transcription was performed on equal volumes of total RNA using the TaqMan microRNA Reverse Transcription Kit and a pool of miRNA‐specific RT primers for hsa‐miR‐122, hsa‐miR‐192, hsa‐miR‐155 and hsa‐miR‐130b (Thermo Fisher). Quantitative real‐time PCR was performed in triplicate using TaqMan microRNA Assays and TaqMan Universal Master Mix II, no UNG (Thermo Fisher) on a Bio‐Rad CFX96 Touch Real‐Time PCR system (Hercules, CA). Prior to RNA isolation, each sample was spiked with cel‐39 (Qiagen), a quantified synthetic miRNA that was used for normalisation. Each analyte was expressed relative to the corresponding cel‐39 measurement in the sample. Relative expression was calculated using the 2−^ΔΔCt^ method and expressed as a ratio to the average value in the NW group [35].

### 2.5 Liver Histology and Analyses

Patients in the MASLD group underwent a liver biopsy as part of their standard clinical care to assess the presence of standard histological features, including steatosis and fibrosis, and to exclude other underlying liver diseases. The liver biopsy was performed using an automatic core 16‐gauge needle (BARD Monopty) under general anaesthesia and ultrasound guidance. Unfragmented samples with a length including at least six complete portal tracts were considered adequate for the study. One biopsy core was formalin fixed, embedded in paraffin, and then stained with haematoxylin–eosin (H&E), Periodic Acid–Schiff (PAS), PAS with diastase (PASD), iron, reticulin and trichrome for the evaluation of histological features by two board‐certified paediatric pathologists. Liver biopsy features for each case were evaluated for steatosis, the MASLD activity score (MAS) and fibrosis stage [3].

A second biopsy core was immediately frozen in liquid nitrogen and stored at −80°C until RNA analyses were performed. The following analyses were performed on samples from 21 patients with MASLD and 1 control participant with obesity who had sufficient liver tissue for analyses. For two participants in the MASLD group, these analyses were not performed because the available liver specimen was too small or damaged. Total RNA was isolated from each liver sample (~10 mg) using the Qiagen miRNeasy Mini Kit (Germantown, MD) and quantified on a NanoDrop ND‐1000 spectrophotometer (Thermo Fisher). After reverse transcription, quantitative PCR was performed in triplicate [36]. The same miRNAs measured in serum (miR‐122, miR‐130b, miR‐155 and miR‐192) were measured in the liver samples, with the non‐coding small nuclear RNA, RNU48, used as an endogenous control. Each analyte was normalised to RNU48 in the sample, the 2−^ΔΔCt^ value was calculated, and then expressed as a ratio to the average value of all samples. Five mRNA transcripts were measured using TaqMan primers (Thermo Fisher): *AGPAT1, DGAT1*, adipose triglyceride lipase (*ATGL*), *SREBP‐1c* and carbohydrate response element binding protein (*ChREBP*). Quantitative PCR was performed using TaqMan Gene Expression Assays and Multiplex Master Mix (Thermo Fisher). Values for each mRNA were normalised to 18S rRNA within the same sample as an endogenous control using multiplexed reactions. Final abundance values were expressed as a ratio to the average value of all samples.

### 2.6 Data Analysis

For comparisons among groups, a Brown‐Forsythe test was used to determine equality of variances. Group comparisons were then performed using either analysis of variance and Tukey's post hoc tests for multiple comparisons, or a Kruskal‐Wallis test and Dunn's tests for post hoc comparisons, as appropriate. The adjusted *p*‐values are presented for all comparisons among groups. Chi‐square tests were used to check for proportional differences between groups for sex and race/ethnicity. Pearson's correlations were used to determine the association between the miRNA and mRNA variables and the descriptive variables. Variables with unequal variances were log‐transformed prior to this analysis. Bonferroni corrections were applied for multiple comparisons. Receiver operator characteristic curves were generated to test how well selected serum biomarkers distinguished the MASLD group from the control groups. A *p*‐value < 0.05 was considered statistically significant for all tests.

## 3 Results

One participant who completed evaluation for MASLD was determined to have normal liver histology, so he was moved into the Ob group. His liver tissue results were included in the liver analyses. As shown in Table 1, the groups did not differ significantly in age, ratio of girls and boys, or race/ethnicity. The MASLD and Ob groups did not differ in body size or composition, and both were larger than the NW group. The Ob group had 5, 14 and 3 participants with Class 1, 2, or 3 obesity, respectively, while the MASLD group had 5, 11 and 5 with Class 1, 2, or 3 obesity, respectively. For the physical activity results, 1 NW, 3 Ob and 2 MASLD participants had missing data because they did not have sufficient wear time. For those who had adequate data for analysis, the average data availability was 7, 7 and 6 days of wear for the NW, Ob and MASLD groups, respectively, with a mean ± standard deviation of 13.9 ± 2.3, 14.0 ± 2.0 and 12.9 ± 1.9 h per day of active use, respectively. Results from the aerobic fitness test were excluded for 1 NW, 3 Ob and 2 MASLD participants because they did not provide a maximal effort (e.g., inability or unwillingness to continue the past a moderate intensity, stated breathing limitation, or leg soreness). For the participants who completed the physical activity and aerobic fitness tests, there were no differences among groups for those variables. There were also no group differences for glucose, HbA1c, total cholesterol, or LDL‐C. The MASLD group had the highest values for triglycerides, NEFA, ALT and AST. Fasting insulin concentration, insulin resistance and C‐reactive protein were higher while HDL‐C was lower in both the Ob and MASLD groups compared to the NW group. Within the MASLD group, 69% of patients had MASH based on MASLD Activity Score ≥ 4, while 22% had borderline MASH and 9% had steatosis without MASH. Fibrosis was absent in 22% of the MASLD group, with the remaining 78% having stage 1 to 3 fibrosis.

**TABLE 1:** Participant characteristics.

	NW control	Ob control	MASLD	*p*
Female/male (count)	9/15	13/11	8/15	0.462
Age	15.2 ± 2.4	16.1 ± 2.5	15.2 ± 2.0	0.683
Race/Ethnicity (count)				
Hispanic	15	12	14	0.692
White	11	14	7	
Native American	4	7	8	
Black	1	3	1	
Asian	2	0	1	
BMI (*z*‐score)	0.11 ± 0.69	2.05 ± 0.33*	2.18 ± 0.33*	< 0.001
Waist circumference (cm)	74.8 ± 8.8	99.6 ± 12.3*	106.6 ± 13.1*	< 0.001
Lean soft tissue (kg)	34.4 ± 8.1	45.5 ± 11.2*	44.2 ± 8.5*	< 0.001
Body fat (kg)	16.0 [12.1, 18.8]	34.6 [27.6, 43.1]*	38.1 [31.4, 47.5]*	< 0.001
Body fat (%)	30.0 ± 5.5	43.7 ± 7.2*	45.6 ± 6.5*	< 0.001
Trunk fat (kg)	7.8 [5.3, 9.4]	18.3 [14.4, 24.5]*	22.2 [17.6, 27.4]*	< 0.001
Trunk fat (%)	28.7 ± 7.1	45.3 ± 7.5*	49.2 ± 6.7*	< 0.001
Physical activity (steps/d)	8537 ± 3420	8243 ± 2913	6914 ± 1885	0.168
VO_2_peak (L/min)	1.96 ± 0.32	1.91 ± 0.31	1.80 ± 0.30	0.194
Glucose (mmol/L)	4.3 ± 0.4	4.3 ± 0.7	4.7 ± 0.9	0.111
Insulin (pmol/L)	53.6 [30.3, 83.1]	107.9 [60.1, 149.0]*	81.5 [48.3, 323.0]*	0.008
iHOMA2‐IR	1.11 ± 0.64	2.39 ± 2.13*	2.95 ± 2.57*	0.001
HbA1c (%)	5.4 ± 0.3	5.4 ± 0.5	5.6 ± 0.3	0.160
Triglycerides (mmol/L)	0.95 ± 0.40	1.30 ± 0.73	2.23 ± 1.36*^†^	< 0.001
Cholesterol (mmol/L)	4.29 ± 0.76	4.22 ± 1.09	4.53 ± 1.08	0.501
HDL‐C (mmol/L)	1.59 ± 0.35	1.25 ± 0.34*	1.02 ± 0.30*	0.001
LDL‐C (mmol/L)	2.39 ± 0.75	2.55 ± 0.89	2.89 ± 0.95	0.591
NEFA (mmol/L)	0.37 ± 0.16	0.39 ± 0.18	0.58 ± 0.26*^†^	< 0.001
ALT (U/L)	19 ± 10	25 ± 13	83 ± 72*^†^	< 0.001
AST (U/L)	32 ± 22	32 ± 14	70 ± 46*^†^	< 0.001
C‐reactive protein (mg/L)	0.19 [0.12, 0.36]	2.8 [1.4, 4.3]*	2.1 [0.95, 3.84]*	< 0.001

*Note:* Results presented as participant counts for sex and race/ethnicity, mean ± standard deviation for normally‐distributed variables, or median [upper, lower interquartile range] for variables with unequal variances. *p*‐Values shown for the main effect of group assessed with a one‐way analysis of variance or Kruskal–Wallis test, except for sex distribution and race/ethnicity, where Chi‐square tests were used. Post hoc differences between groups are designated as * different from NW, *p* < 0.05; ^†^ different from Ob, *p* < 0.05 after adjusting for multiple comparisons. Race/ethnicity compositions exceed 100% because there were 7 or 8 participants in each group who identified as 2 or more categories. Values for physical activity and aerobic fitness tests are for 23 NW controls, 21 Ob controls and 21 participants with MASLD.

Abbreviations: ALT, alanine aminotransferase; AST, aspartate aminotransferase; BMI, body mass index; HDL‐C, high‐density lipoprotein cholesterol; iHOMA2‐IR, interactive homeostatic model of assessment for insulin resistance; LDL‐C, low‐density lipoprotein cholesterol; NEFA, nonesterified fatty acids; VO_2_peak, peak oxygen uptake (values shown are adjusted for lean soft tissue mass).

**FIGURE 1:**
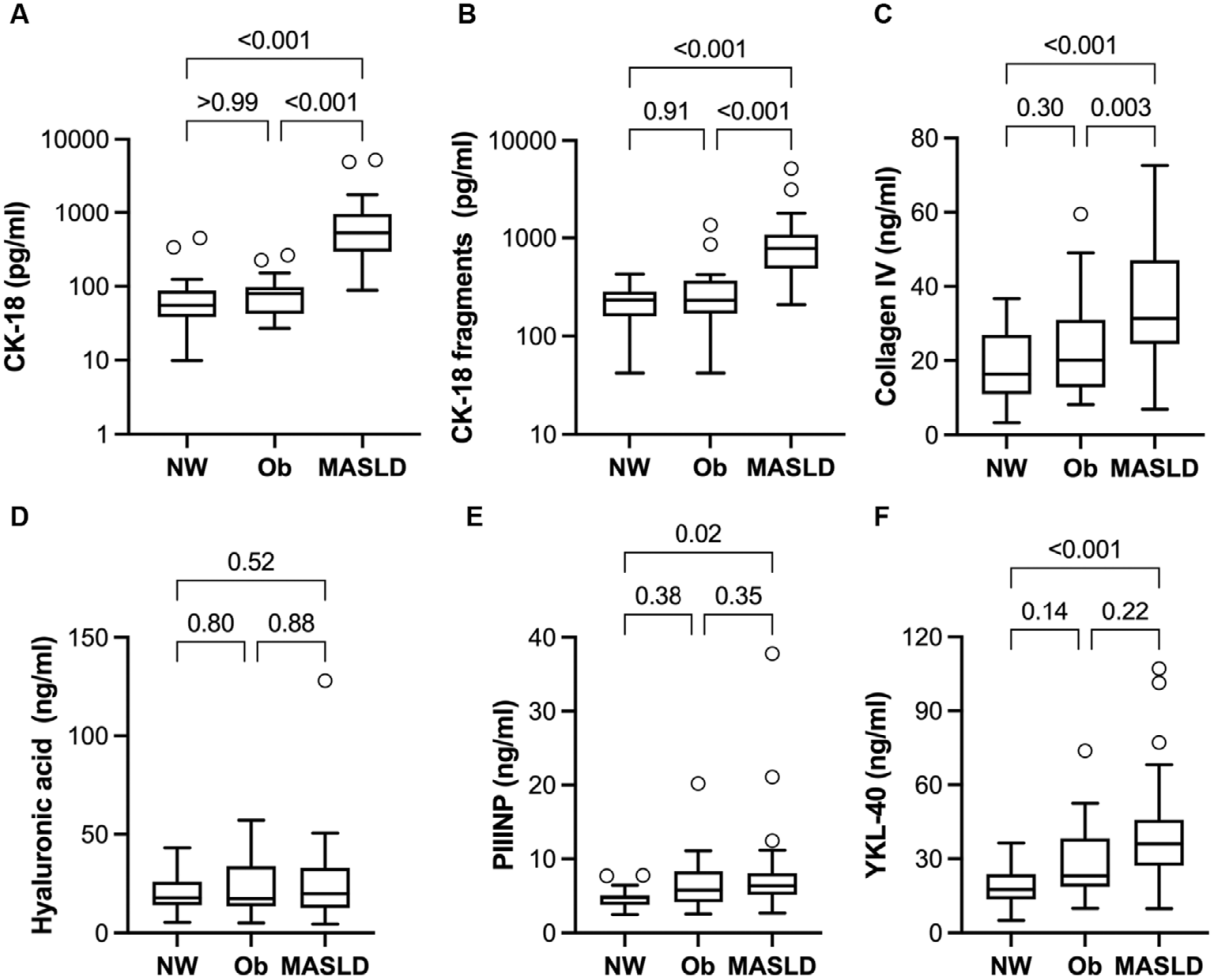
Serum concentrations of (A) CK‐18, (B) CK‐18 fragments, (C) collagen IV, (D) hyaluronic acid, (E) PIIINP and (F) YKL‐40 in control participants with either normal weight (NW, *n* = 24) or obesity (Ob, *n* = 24), and participants with metabolic dysfunction associated liver disease (MASLD, *n* = 23). Comparisons between groups are *p*‐values adjusted for multiple comparisons. Boxes show the median and interquartile range for each group. Error bars show 1.5× the interquartile range, and circles beyond those lines are considered outliers. The *y*‐axes for panels A and B are on a log scale for better clarity. Comparisons between groups are *p*‐values adjusted for multiple comparisons.

The protein biomarkers of MASLD are shown in Figure 1. The MASLD group had higher average values than both control groups for CK‐18 (8.5‐ and 8.3‐fold higher than the NW and Ob groups, respectively), CK‐18 fragments (3.7‐ and 2.4‐fold higher than the NW and Ob groups, respectively) and Col‐IV (1.0‐ and 0.6‐fold higher than the NW and Ob groups, respectively). PIIINP and YKL‐40 were higher in the MASLD group than the NW group (81% and 110%, respectively) but not significantly different between the MASLD and Ob groups. There were no differences among groups in hyaluronic acid.

**FIGURE 2:**
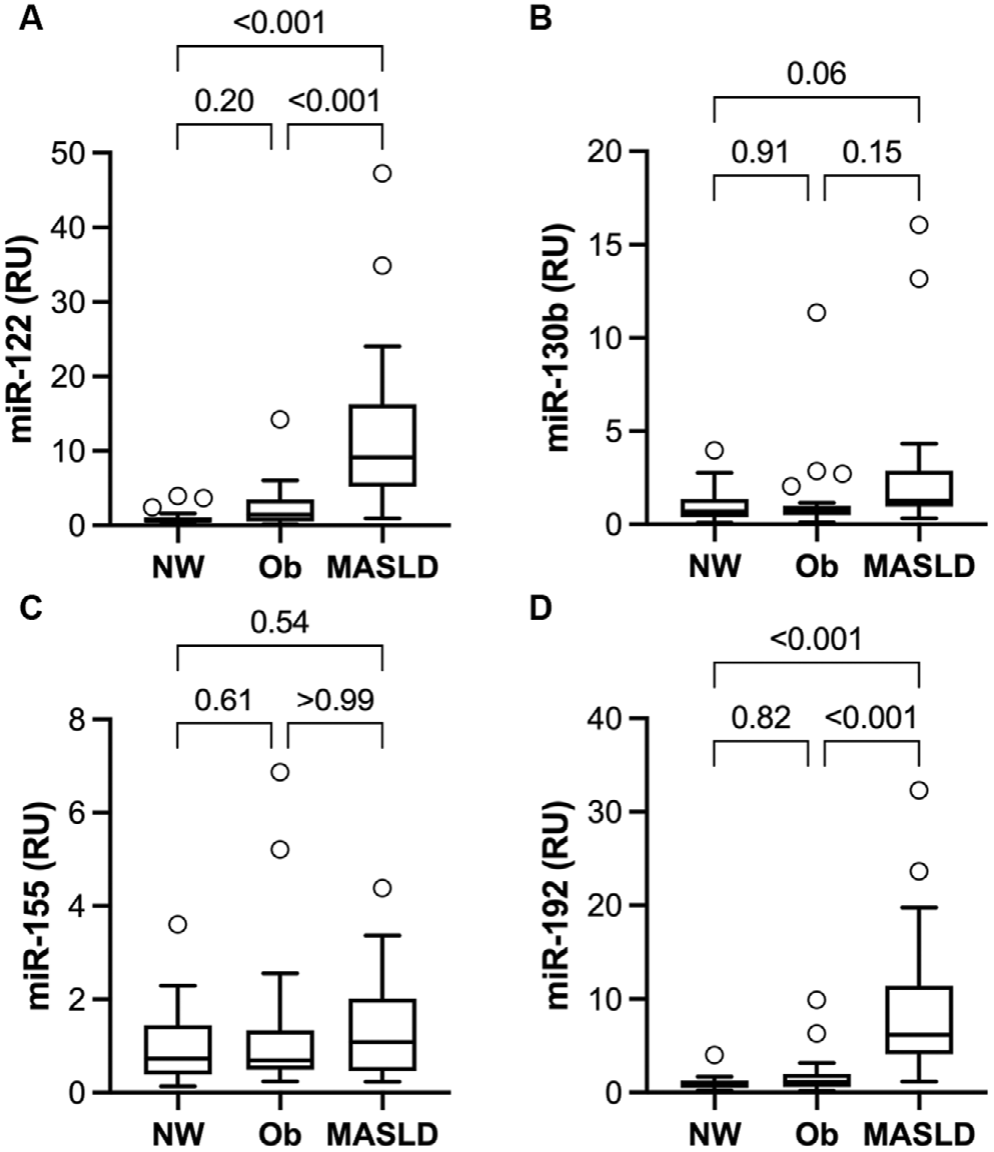
Serum concentrations of (A) miR‐122, (B) miR‐130b, (C) miR‐155 and (D) miR‐192 in control participants with either normal weight (NW, *n* = 24) or obesity (Ob, *n* = 24), and participants with metabolic dysfunction‐associated liver disease (MASLD, *n* = 23). Boxes show the median and interquartile range for each group. Error bars show 1.5× the interquartile range, and circles beyond those lines are considered outliers. Comparisons between groups are *p*‐values adjusted for multiple comparisons.

Serum miRNAs are shown in Figure 2. Average miR‐122 was 11‐ and 6.5‐fold higher and miR‐192 was 8.8‐ and 5.5‐fold higher in the MASLD group versus the NW and Ob groups, respectively. Serum miR‐130b was increased in the MASLD group ~1.6‐fold versus the NW group but did not reach statistical significance. Serum miR‐155 did not differ among groups.

**FIGURE 3:**
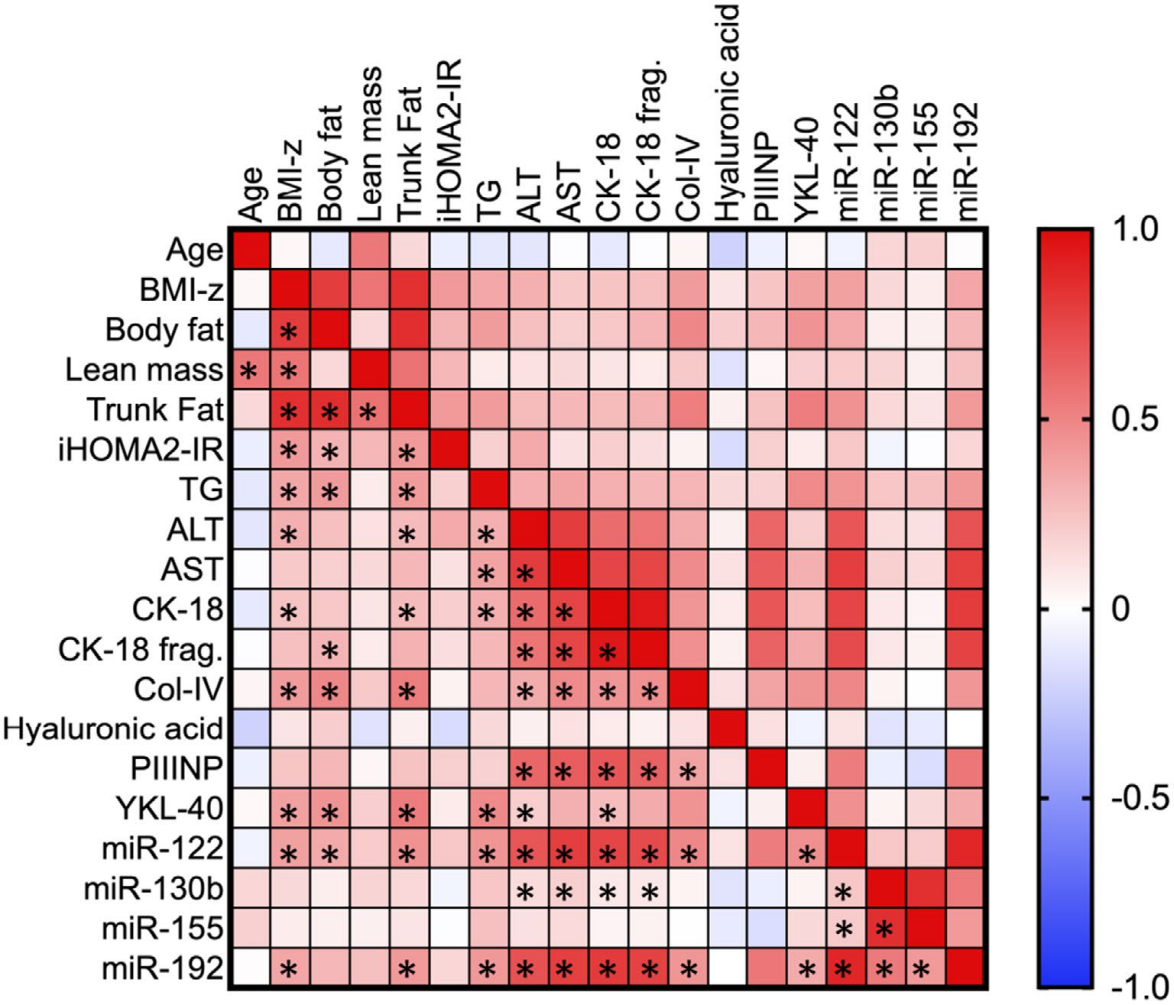
Correlation matrix among participant descriptive variables and serum variables. Skewed variables were log‐transformed before analysis. Colour coding of each pairwise correlation follows the legend on the right for direction and magnitude. Asterisks in the lower half of the grid denote correlations that were significant after Bonferroni correction for multiple comparisons.

**FIGURE 4:**
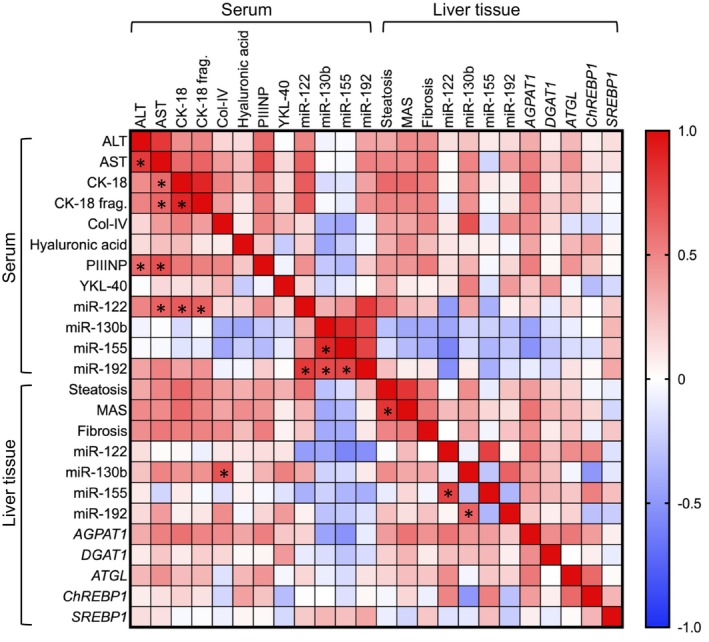
Correlation matrix among variables measured in serum and liver biopsies. Skewed variables were log‐transformed before analysis. Colour coding of each pairwise correlation follows the legend on the right for direction and magnitude. Asterisks in the lower half of the grid denote correlations that were significant after Bonferroni correction for multiple comparisons.

**FIGURE 5:**
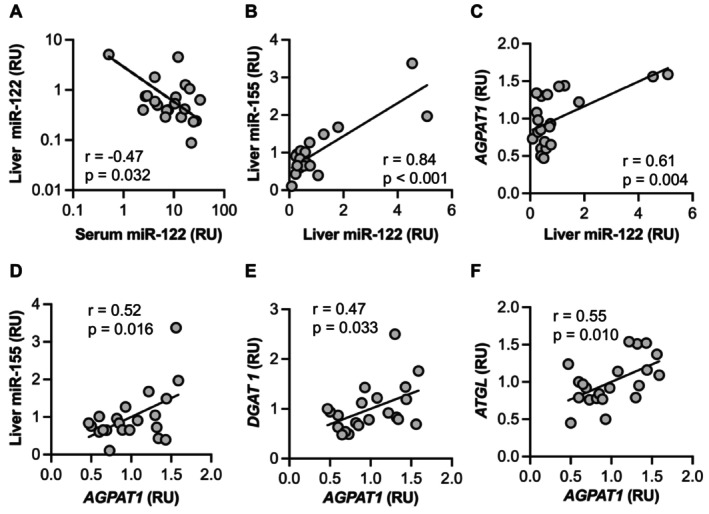
Correlations among selected results for liver variables. (A–C) Liver miR‐122 abundance was inversely associated with serum miR‐122 and positively associated with liver miR‐155, liver AGPAT1 mRNA and liver *DGAT1* mRNA. (D–F) Liver *AGPAT1* mRNA was positively associated with liver miR‐155 abundance, liver *DGAT1* mRNA and liver *ATGL* mRNA. Results shown for 21 patients with MASLD and 1 control participant with obesity who had liver tissue available for analysis. Unadjusted *p*‐values for the Pearson correlation coefficients are shown. The threshold for significance after Bonferroni correction is 0.0023.

A correlation matrix (Figure 3) that included descriptive and serum variables for the whole cohort revealed several significant associations between the protein and miRNA biomarkers and other variables. Notably, serum ALT, CK‐18, Col‐IV, YKL‐40, miR‐122 and serum miR‐192 were all positively correlated with trunk fat, and most were positively correlated with one another. A second correlation matrix (Figure 4) was created to identify associations between serum biomarkers and the measurements made in liver biopsies, including the histology scores, miRNAs and mRNAs. After controlling for multiple comparisons, two notable findings were positive correlations between liver abundance of miR‐122 and miR‐155 (*r* = 0.84, shown in Figure 5) and liver abundance of miR‐130b and miR‐192 (*r* = 0.64). In this analysis, none of the biomarkers were significantly correlated with steatosis grade, MAS, or fibrosis stage, and there were only a few other correlations that remained significant after Bonferroni correction. Notable correlations with histological scores that approached the corrected significance threshold included steatosis and CK‐18 (*r* = 0.58), CK‐18 fragments (*r* = 0.56) and serum miR‐122 (*r* = 0.55). MASLD Activity Score was also positively associated with CK‐18 (*r* = 0.60), CK‐18 fragments (*r* = 0.58) and negatively associated with serum miR‐130b (*r* = 0.54) and serum miR‐155 (*r* = 0.53) while fibrosis was positively associated with PIIINP (*r* = 0.58), AST (r = 0.53) and CK‐18 fragments (r = 0.53), and negatively associated with serum miR‐155 (*r* = −0.52). All these correlations had an unadjusted *p*‐value < 0.015.

Figure 5 shows some of the correlations of variables measured in liver biopsies. Of the four miRNAs measured, only miR‐122 had a significant (negative) correlation between its content in serum and liver abundance. Liver miR‐122 was also positively correlated with liver miR‐155 and *AGPAT1* mRNA. Another notable finding was that *AGPAT1* mRNA abundance was positively correlated with liver miR‐155 and mRNAs for *DGAT1* and *ATGL*.

**FIGURE 6:**
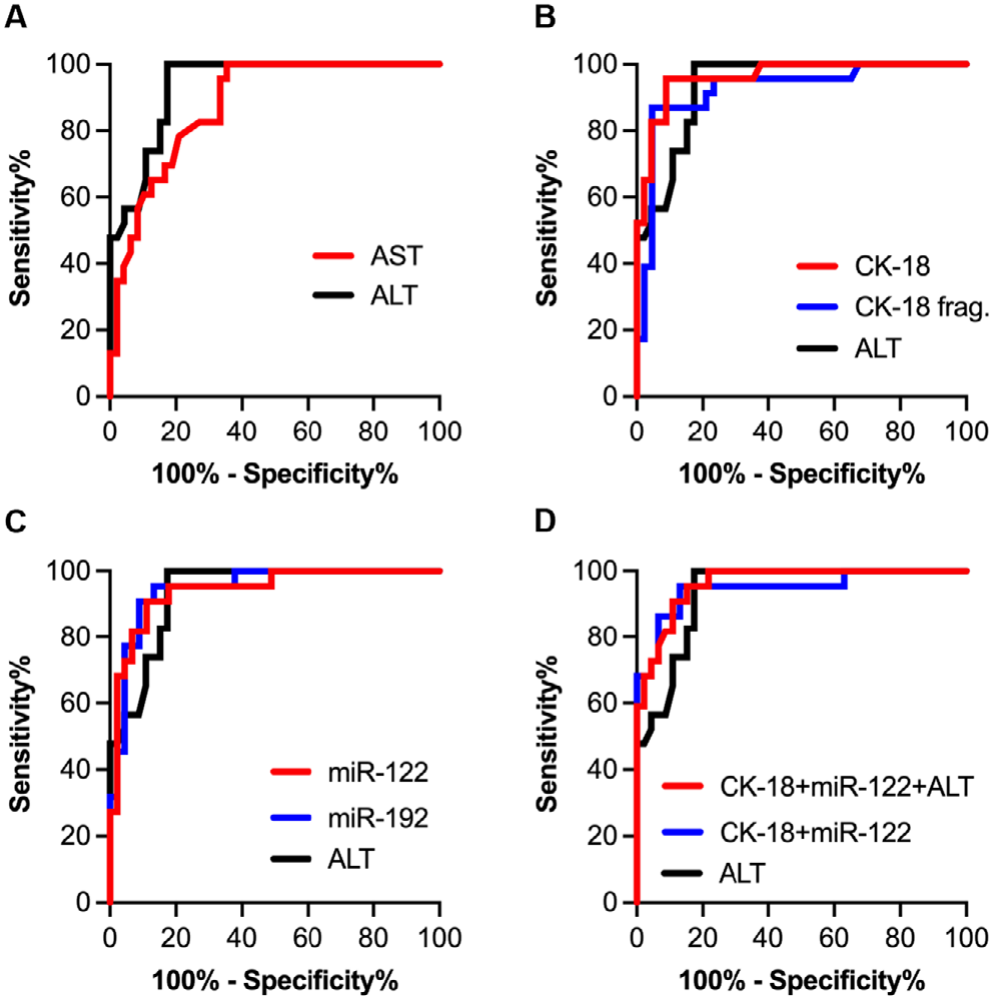
Receiver operator characteristic (ROC) curves to determine how selected serum biomarkers perform in identifying MASLD cases from controls. (A) Serum AST had a lower area under the ROC curve (AUROC, 0.878) than ALT (0.935). (B) Compared to ALT, AUROC for serum CK‐18 (0.962) was higher, while serum CK‐18 fragments were lower (0.925). (C) AUROC for serum miR‐122 (0.944) and serum miR‐192 (0.945) were both greater than ALT. (D) Combined values for serum CK‐18 and serum miR‐122 resulted in AUROC = 0.949. Combining values for ALT, serum CK‐18 and serum miR‐122 raised the AUROC to 0.963.

Physical activity (daily step count) and aerobic fitness were not significantly associated with any of the serum or liver biomarkers. Indices of adiposity, including BMI‐z, body fat and trunk fat were modestly, positively associated with a few biomarkers, with the strongest correlations between trunk fat (in kg) and liver miR‐130b (*r* = 0.54), serum collagen IV (*r* = 0.53), serum YKL‐40 (*r* = 0.53) and serum miR‐122 (*r* = 0.45). To determine how well the serum biomarkers distinguish participants with MASLD from those without, ROC curves were constructed for variables that were significantly increased in the MASLD group compared to one or both control groups (Figure 6). The AUROC for ALT was 0.935, while values for CK‐18 fragments (0.925), AST (0.878), YKL‐40 (0.735), PIIINP (0.687) were lower. The AUROC values for miR‐122 (0.942), miR‐192 (0.948) and CK‐18 (0.962) were higher than ALT. Combining the values for CK‐18, miR‐122 and ALT (after normalising each by expressing individual values relative to the mean of the NW group) resulted in an AUROC of 0.963. No other combination of these serum biomarkers resulted in a higher AUROC.

## 4 Discussion

We compared multiple protein and miRNA biomarkers of MASLD and liver fibrosis in a paediatric cohort, and included liver tissue values for histopathology, miRNAs and mRNAs involved in lipid metabolism. We found that CK‐18, miR‐122 and miR‐192 performed as well or better than the commonly measured liver enzyme ALT at discriminating cases of MASLD and liver fibrosis, but recognise the need to replicate these findings in a larger cohort that includes more cases with advanced fibrosis.

CK‐18 is the most abundant intermediate filament protein in the liver and its cleavage products are released into the circulation when apoptotic pathways are activated [37]. We found that both the full‐length (M65) and fragmented (M30) CK‐18 in serum were higher in the MASLD group than either control group, consistent with prior reports in children and adolescents [11, 12, 20, 38] and adults [13, 19, 24] with MASLD. Full‐length CK‐18 was the best predictor of MASLD, based on AUROC, among the biomarkers measured in the current study. In a study of adults, Feldstein et al. [13] reported that CK‐18 detected MASH with over 90% specificity, while studies in children found that CK‐18 fragments could identify liver fibrosis [12, 39]. In a review of MASLD biomarkers Nobili et al. [40] stated that the full‐length CK‐18 may identify MASH, but is not yet established as a general screening biomarker for MASLD. Those authors noted that CK‐18 reflects hepatocellular apoptosis, but does not consistently reflect the severity of MASH in children and adolescents [40].

In comparison to CK‐18, the other protein biomarkers in serum were not as good at differentiating patients with MASLD from controls or correlating with liver features. We measured three markers of fibrosis that, like CK‐18, reflect liver apoptosis and turnover of the extracellular matrix. Collagen IV is a basement membrane protein, PIIINP is generated during turnover of collagen III, and hyaluronic acid is a glycosaminoglycan. We also measured a marker of inflammation, YKL‐40, a glycoprotein that is produced by several cell types, including macrophages [9]. Of these, neither PIIINP nor hyaluronic acid differed between the MASLD and Ob groups, unlike previous reports showing that they were increased in patients with MASLD [9, 10]. In contrast, Col‐IV and YKL‐40 were higher in the MASLD group than both control groups, and both were positively correlated with measures of adiposity (BMI‐z, body fat and trunk fat), ALT, and CK‐18. However, neither was correlated with liver fibrosis or MAS, and they had lower AUROCs than ALT, so their potential value as biomarkers was not confirmed. To our knowledge, values for Col‐IV in adolescents with MASLD have not been previously reported. In adults, serum Col‐IV was shown to increase with the severity of MASH and predicted the presence of severe fibrosis [9, 14]. As reflected in our cohort and some others [41], advanced fibrosis is far less common in adolescents with MASLD than in adults, which may help explain why some extracellular matrix proteins show minimal or modest association with fibrosis stage in paediatric patients. Additionally, the inherently higher tissue turnover in children during growth may limit the usefulness of structural proteins as biomarkers for fibrosis. For example, Cohen et al. [42] found that a serum marker of collagen III degradation, PRO‐C3, was elevated in children and adolescents with MASLD and advanced fibrosis. However, PRO‐C3 also increased with age and bone growth markers, which they argued could reduce the specificity of this biomarker until late adolescence or early adulthood.

We found that serum miR‐122 and miR‐192 were increased in patients with MASLD compared to both control groups, while miR‐130b and miR‐155 did not differ among groups. Notably, both miR‐122 and ‐192 were strongly correlated with ALT but had higher AUROC values than ALT, supporting the need for further validation of these biomarkers in the paediatric population. In prior studies of children or adolescents, miR‐122 was increased in some, but not all participants with MASLD [20, 22] and associated with liver fibrosis stage [21]. In other studies of children, serum miR‐122 was increased with obesity and insulin resistance but liver health was not determined, so unrecognised liver pathology may be responsible for the findings in those studies [43, 44]. Increased miR‐122 has been reported in adults with MASLD [19, 24, 28, 45] and in some studies miR‐122 distinguished MASH from uncomplicated steatosis [19, 24, 28]. Rodent and hepatocyte culture studies provided evidence that the liver increases production of miR‐122 when fatty acids rise, which may in turn slow hepatic lipogenesis through the inhibition of triglyceride synthesis enzymes like *AGPAT1* and *DGAT1* [16, 26]. We did not find that either serum or liver miR‐122 content was correlated to the abundance of transcripts for *AGPAT1* and *DGAT1* in the liver. As MASLD progresses, studies in hepatocytes [16], rodent models of liver disease [16, 25], and adults [19, 26, 45] support the current understanding that the export of miR‐122 increases. Our finding, for the first time in adolescents, that serum and liver abundance of miR‐122 are inversely correlated is consistent with that idea. The reason that miR‐122 export increases is not yet clear, but during another type of nutrient stress (amino acid starvation) the liver upregulates a protein that stabilises miR‐122 and directs it toward export [46], so a similar mechanism may occur during the lipid and/or inflammatory stress of MASLD. Like miR‐122, miR‐192 is abundant in liver, although it is also produced in other tissues [47]. It was reported to be increased with MASLD in adults [19, 24, 27, 28] but in only one prior study of children and adolescents [22]. The study by Lischka et al. [22] was performed on paediatric patients with severe obesity, a subset of whom were determined to have MASLD based on hepatic fat fraction. They found that miR‐192 was weakly correlated with markers of inflammation.

Neither miR‐155 or miR‐130b serum concentrations were affected by MASLD. We measured miR‐155 because it was shown in adults with MASLD to be lower in serum and liver compared to controls without MASLD [29]. miR‐155 is also produced by adipose tissue where it activates macrophages, resulting in inflammation and insulin resistance in hepatocytes and adipocytes [30, 31]. A reduction in liver miR‐155 may contribute to hepatic lipogenesis since one of its transcriptional targets, *SREBP1*, an activator of fatty acid synthesis, is increased in liver from patients with MASLD [29]. We found that liver miR‐155 was positively correlated with miR‐122 and *AGPAT1*, but there was no association with *SREBP1*. It is possible that extrahepatic expression, particularly from adipose and immune cells, diminishes the specificity of miR‐155 as a circulating liver biomarker. Furthermore, developmental differences in immune activity during adolescence may influence miR‐155 expression or release patterns. Serum miR‐130b in the MASLD group was slightly, but non‐significantly, higher than the control groups and positively correlated with ALT, AST, CK‐18, CK‐18 fragments, miR‐122 and miR‐155. These results suggest that miR‐130b modestly reflects the presence of liver inflammation and injury, but not as strongly as the other biomarkers evaluated. Liver miR‐130b abundance appears to support the development of steatosis. In a rodent high‐fat feeding model, miR‐130b was increased in the liver, but its suppression resulted in lower hepatic steatosis and insulin resistance [48]. This may be partially mediated through inhibition of lipid oxidation as Jiang et al. [36] showed that miR‐130b suppresses expression of PPAR‐gamma‐co‐activator‐1‐alpha (PGC1α), a master regulator of mitochondrial oxidative capacity. Our findings suggest that miR‐130b may contribute to the metabolic remodelling seen in MASLD, but its serum levels may not adequately reflect liver pathology, either due to limited export or insufficient assay sensitivity. These results suggest that miR‐130b and miR‐155 may still play pathophysiological roles in MASLD, particularly within hepatic or adipose tissue, but are less promising than miR‐122 or miR‐192 as serum‐based diagnostic biomarkers in adolescents. Further studies evaluating miRNA localization, export mechanisms and age‐dependent regulation may help clarify their utility in paediatric MASLD.

A strength of the current study is the inclusion of well‐defined control groups of adolescents with NW and Ob, allowing differentiation of biomarkers associated with MASLD from those related to obesity alone. This distinction is critical given the increasing prevalence of obesity and its potential adverse effect on liver health in children. We also excluded participants with type 2 diabetes, a condition that often accompanies MASLD, but could confound interpretation of some outcomes. In adults, MASLD is frequently described as a condition that features insulin resistance (often with type 2 diabetes) [49]. In our cohort, both the Ob and MASLD groups had elevated insulin resistance compared to the NW group, but several biomarkers (ALT, AST, NEFA, triglycerides, CK‐18, CK‐18 fragments, collagen IV, miR‐122 and miR‐192) were higher in the MASLD group compared to the Ob group, highlighting that insulin resistance is not a distinguishing feature of MASLD in our study. Our cohort also included participants with a mix of racial/ethnic backgrounds.

Among the limitations are the small sample size and the lack of an independent validation group. There were also few patients with advanced fibrosis, which may also affect the generalisability of our findings. As is typical in this age group, the majority of MASLD patients had mild to moderate fibrosis (stages 1–2), and none presented with cirrhosis [50, 51]. Although patients with MASLD had liver tissue available for histology and measures of miRNA and mRNA, we did not have liver samples from healthy controls for comparison, as such samples are difficult to acquire in a paediatric population. Another challenge of assessing biomarkers for the presence of MASLD or specific MASLD features is specificity. Liver‐derived proteins and miRNAs may increase in circulation due to the presence of liver conditions other than MASLD. We measured ALT using a point‐of‐care analyser and used the reference range proposed by the manufacturer and confirmed in our laboratory. We acknowledge that sex‐specific and age‐specific reference ranges have been proposed in the literature. In particular, in their analysis of results from the National Health and Nutrition Examination Study, Schwimmer et al. [52] suggested upper limits of normal ALT in healthy adolescents as 25.8 U/L for girls and 22.1 U/L for boys. However, for the purposes of our study, which focused on excluding individuals with suspected MASLD from the OB and NW control groups, we used 47 U/L as a conservative clinical threshold consistent with our institutional standards. We found that physical activity did not differ significantly among groups and was not correlated with the primary outcomes. A nutritional survey was not performed so we cannot determine if dietary quality contributed to the variations among groups in the serum and liver biomarkers. Sugar intake in particular is known to have a strong influence on hepatic steatosis in children and adolescents, so it could also impact liver production and export of miRNAs and biomarker proteins like CK‐18 and ALT [53, 54]. Although all participants were confirmed to be at Tanner stage 2 or above, we did not perform specific Tanner staging. Insulin resistance has been shown to vary during puberty [55, 56] so it is possible there are similar variations in the serum or liver proteins and miRNAs that went undetected. Lastly, since this was a cross‐sectional study, we could not determine how the measured biomarkers change over time or reflect disease progression.

In conclusion, the current study shows that the concentrations of serum CK‐18, serum miR‐122 and serum miR‐192 may be as good or slightly better than serum ALT activity at identifying MASLD in paediatric patients. Although serum CK‐18 had the highest individual predictive power among those three biomarkers, serum miR‐122 has promise due to the converging evidence that it is exported from the liver during the progression of MASLD in rodent models, adults, and as we now show, in adolescents. Additional validation in larger cohorts and longitudinal assessments is needed.

## Author Contributions

**Neeharika Bade:** sample analyses and writing – original draft (equal). **Diana A. Hellman:** writing – original draft (equal). **David Matye:** writing – original draft (equal). **Shaoning Jiang:** writing – original draft (equal). **Jeanie B. Tryggestad:** writing – original draft (equal). **Zhongxin Yu:** writing – original draft (equal). **Kevin R. Short:** writing – original draft (equal). **Sirish K. Palle:** writing – original draft (equal).

## Conflicts of Interest

The authors declare no conflicts of interest.

## Data Availability

The data that support the findings of this study are available on request from the corresponding author. The data are not publicly available due to privacy or ethical restrictions.

## References

[1] M. E. Rinella , J. V. Lazarus , V. Ratziu , et al., “A Multisociety Delphi Consensus Statement on New Fatty Liver Disease Nomenclature,” Hepatology 78, no. 6 (2023): 1966–1986, 10.1097/HEP.0000000000000520. 37363821 PMC10653297

[2] A. K. Sahota , W. L. Shapiro , K. P. Newton , S. T. Kim , J. Chung , and J. B. Schwimmer , “Incidence of Nonalcoholic Fatty Liver Disease in Children: 2009‐2018,” Pediatrics 146, no. 6 (2020): e20200771, 10.1542/peds.2020-0771. 33214329 PMC7706110

[3] M. B. Vos , S. H. Abrams , S. E. Barlow , et al., “NASPGHAN Clinical Practice Guideline for the Diagnosis and Treatment of Nonalcoholic Fatty Liver Disease in Children: Recommendations From the Expert Committee on NAFLD (ECON) and the North American Society of Pediatric Gastroenterology, Hepatology and Nutrition (NASPGHAN),” Journal of Pediatric Gastroenterology and Nutrition 64, no. 2 (2017): 319–334, 10.1097/mpg.0000000000001482. 28107283 PMC5413933

[4] A. E. Feldstein and V. Nobili , “Biomarkers in Nonalcoholic Fatty Liver Disease: A New Era in Diagnosis and Staging of Disease in Children,” Journal of Pediatric Gastroenterology and Nutrition 51, no. 4 (2010): 378–379, 10.1097/MPG.0b013e3181ecf3d4. 20808243 PMC2950320

[5] E. M. Brunt , D. E. Kleiner , D. H. Carpenter , et al., “NAFLD: Reporting Histologic Findings in Clinical Practice,” Hepatology 73, no. 5 (2021): 2028–2038, 10.1002/hep.31599. 33111374

[6] A. Wieckowska , A. J. McCullough , and A. E. Feldstein , “Noninvasive Diagnosis and Monitoring of Nonalcoholic Steatohepatitis: Present and Future,” Hepatology 46, no. 2 (2007): 582–589, 10.1002/hep.21768. 17661414

[7] Z. M. Younossi , M. Jarrar , C. Nugent , et al., “A Novel Diagnostic Biomarker Panel for Obesity‐Related Nonalcoholic Steatohepatitis (NASH),” Obesity Surgery 18, no. 11 (2008): 1430–1437, 10.1007/s11695-008-9506-y. 18500507

[8] M. Crespo , S. Lappe , A. E. Feldstein , and N. Alkhouri , “Similarities and Differences Between Pediatric and Adult Nonalcoholic Fatty Liver Disease,” Metabolism 65, no. 8 (2016): 1161–1171, 10.1016/j.metabol.2016.01.008. 26961580

[9] E. Kumagai , Y. Mano , S. Yoshio , et al., “Serum YKL‐40 as a Marker of Liver Fibrosis in Patients With Non‐Alcoholic Fatty Liver Disease,” Scientific Reports 6 (2016): 35282, 10.1038/srep35282. 27739482 PMC5064386

[10] A. Mosca , D. Comparcola , I. Romito , et al., “Plasma N‐Terminal Propeptide of Type III Procollagen Accurately Predicts Liver Fibrosis Severity in Children With Non‐Alcoholic Fatty Liver Disease,” Liver International 39, no. 12 (2019): 2317–2329, 10.1111/liv.14225. 31436362

[11] N. Santoro , A. E. Feldstein , E. Enoksson , et al., “The Association Between Hepatic Fat Content and Liver Injury in Obese Children and Adolescents. Effects of Ethnicity, Insulin Resistance, and Common Gene Variants,” Diabetes Care 36, no. 5 (2013): 1353–1360, 10.2337/dc12-1791. 23275357 PMC3631865

[12] E. Fitzpatrick , R. R. Mitry , A. Quaglia , M. J. Hussain , R. DeBruyne , and A. Dhawan , “Serum Levels of CK18 M30 and Leptin Are Useful Predictors of Steatohepatitis and Fibrosis in Paediatric NAFLD,” Journal of Pediatric Gastroenterology and Nutrition 51, no. 4 (2010): 500–506, 10.1097/MPG.0b013e3181e376be. 20808246

[13] A. E. Feldstein , A. Wieckowska , A. R. Lopez , Y. C. Liu , N. N. Zein , and A. J. McCullough , “Cytokeratin‐18 Fragment Levels as Noninvasive Biomarkers for Nonalcoholic Steatohepatitis: A Multicenter Validation Study,” Hepatology 50, no. 4 (2009): 1072–1078, 10.1002/hep.23050. 19585618 PMC2757511

[14] M. Mizuno , T. Shima , H. Oya , et al., “Classification of Patients With Non‐Alcoholic Fatty Liver Disease Using Rapid Immunoassay of Serum Type IV Collagen Compared With Liver Histology and Other Fibrosis Markers,” Hepatology Research 47, no. 2 (2017): 216–225, 10.1111/hepr.12710. 26997642

[15] C. J. Pirola and S. Sookoian , “MicroRNAs as Messengers of Liver Diseases: Has the Message Finally Been Decrypted?,” Clinical Science (London, England) 136, no. 5 (2022): 323–328, 10.1042/cs20211177. 35234251

[16] C. Chai , M. Rivkin , L. Berkovits , et al., “Metabolic Circuit Involving Free Fatty Acids, microRNA 122, and Triglyceride Synthesis in Liver and Muscle Tissues,” Gastroenterology 153, no. 5 (2017): 1404–1415, 10.1053/j.gastro.2017.08.013. 28802563

[17] J. Yu , J. Peng , Z. Luan , F. Zheng , and W. Su , “MicroRNAs as a Novel Tool in the Diagnosis of Liver Lipid Dysregulation and Fatty Liver Disease,” Molecules 24 (2019): 230, 10.3390/molecules24020230. 30634538 PMC6358728

[18] G. N. López‐Sánchez , M. Dóminguez‐Pérez , M. Uribe , N. C. Chávez‐Tapia , and N. Nuño‐Lámbarri , “Non‐Alcoholic Fatty Liver Disease and microRNAs Expression, How It Affects the Development and Progression of the Disease,” Annals of Hepatology 21 (2021): 100212, 10.1016/j.aohep.2020.04.012. 32533953

[19] C. J. Pirola , T. Fernández Gianotti , G. O. Castaño , et al., “Circulating microRNA Signature in Non‐Alcoholic Fatty Liver Disease: From Serum Non‐Coding RNAs to Liver Histology and Disease Pathogenesis,” Gut 64, no. 5 (2015): 800–812, 10.1136/gutjnl-2014-306996. 24973316 PMC4277726

[20] S. Brandt , J. Roos , E. Inzaghi , et al., “Circulating Levels of miR‐122 and Nonalcoholic Fatty Liver Disease in Pre‐Pubertal Obese Children,” Pediatric Obesity 13 (2017): 175–182, 10.1111/ijpo.12261. 29271122

[21] Y. J. Li , B. O. Baumert , N. Stratakis , et al., “Circulating microRNA Expression and Nonalcoholic Fatty Liver Disease in Adolescents With Severe Obesity,” World Journal of Gastroenterology 30, no. 4 (2024): 332–345, 10.3748/wjg.v30.i4.332. 38313232 PMC10835537

[22] J. Lischka , A. Schanzer , A. Hojreh , et al., “Circulating microRNAs 34a, 122, and 192 Are Linked to Obesity‐Associated Inflammation and Metabolic Disease in Pediatric Patients,” International Journal of Obesity 45, no. 8 (2021): 1763–1772, 10.1038/s41366-021-00842-1. 33986456 PMC8310785

[23] J. Chang , E. Nicolas , D. Marks , et al., “miR‐122, a Mammalian Liver‐Specific microRNA, Is Processed From Hcr mRNA and May Downregulate the High Affinity Cationic Amino Acid Transporter CAT‐1,” RNA Biology 1, no. 2 (2004): 106–113, 10.4161/rna.1.2.1066. 17179747

[24] P. P. Becker , M. Rau , J. Schmitt , et al., “Performance of Serum microRNAs ‐122, ‐192 and ‐21 as Biomarkers in Patients With Non‐Alcoholic Steatohepatitis,” PLoS One 10, no. 11 (2015): e0142661, 10.1371/journal.pone.0142661. 26565986 PMC4643880

[25] D. Povero , A. Eguchi , H. Li , et al., “Circulating Extracelluar Vesicles With Specific Proteome and Liver microRNAs Are Potential Biomarkers for Liver Injury in Experimental Fatty Liver Disease,” PLoS One 9, no. 12 (2014): e113651, 10.1371/journal.pone.0113651. 25470250 PMC4254757

[26] O. Cheung , P. Puri , C. Eicken , et al., “Nonalcoholic Steatohepatitis Is Associated With Altered Hepatic MicroRNA Expression,” Hepatology 48, no. 6 (2008): 1810–1820, 10.1002/hep.22569. 19030170 PMC2717729

[27] X. L. Liu , P. Q , H. X. Cao , et al., “Lipotoxic Hepatocyte‐Derived Exosomal miR‐192‐5p Activates Macrophages via Rictor/Akt/FoxO1 Signaling in NAFLD,” Hepatology 72, no. 2 (2020): 454–469, 10.1002/hep.31050. 31782176 PMC10465073

[28] Y. Tan , G. Ge , T. Pan , D. Wen , and J. Gan , “A Pilot Study of Serum microRNAs Panel as Potential Biomarkers for Diagnosis of Nonalcoholic Fatty Liver Disease,” PLoS One 9, no. 8 (2014): e105192, 10.1371/journal.pone.0105192. 25141008 PMC4139327

[29] L. Wang , N. Zhang , Z. Wang , D. Ai , Z. Cao , and H. Pan , “Decreased miR‐155 Level in the Peripheral Blood of Non‐Alcoholic Fatty Liver Disease Patients May Serve as a Biomarker and May Influence LXR Activity,” Cellular Physiology and Biochemistry 39, no. 6 (2016): 2239–2248, 10.1159/000447917. 27832630

[30] M. A. Mori , R. G. Ludwig , R. Garcia‐Martin , B. B. Brandão , and C. R. Kahn , “Extracellular miRNAs: From Biomarkers to Mediators of Physiology and Disease,” Cell Metabolism 30, no. 4 (2019): 656–673, 10.1016/j.cmet.2019.07.011. 31447320 PMC6774861

[31] J. B. Tryggestad , A. M. Teague , D. P. Sparling , S. Jiang , and S. D. Chernausek , “Macrophage‐Derived microRNA‐155 Increases in Obesity and Influences Adipocyte Metabolism by Targeting Peroxisome Proliferator‐Activated Receptor Gamma,” Obesity (Silver Spring) 27, no. 11 (2019): 1856–1864, 10.1002/oby.22616. 31531958 PMC6832842

[32] R. J. Kuczmarski , C. L. Ogden , S. S. Guo , et al., “2000 CDC Growth Charts for the United States: Methods and Development,” Vital and Health Statistics 11, no. 246 (2002): 1–190. 12043359

[33] N. R. Hill , J. C. Levy , and D. R. Matthews , “Expansion of the Homeostasis Model Assessment of B‐Cell Function and Insulin Resistance to Enable Clinical Trial Outcome Modeling Through the Interactive Adjustment of Physiology and Treatment: iHOMA2,” Diabetes Care 36 (2013): 2324–2330. 23564921 10.2337/dc12-0607 PMC3714535

[34] M. Sampson , C. Ling , Q. Sun , et al., “A New Equation for Calculation of Low‐Density Lipoprotein Cholesterol in Patients With Normolipidemia and/or Hypertriglyceridemia,” JAMA Cardiology 5, no. 5 (2020): 540–548, 10.1001/jamacardio.2020.0013. 32101259 PMC7240357

[35] K. J. Livak and T. D. Schmittgen , “Analysis of Relative Gene Expression Data Using Real‐Time Quantitative PCR and the 2(‐Delta Delta C(T)) Method,” Methods 25, no. 4 (2001): 402–408, 10.1006/meth.2001.1262. 11846609

[36] S. Jiang , A. M. Teague , J. B. Tryggestad , and S. D. Chernausek , “Role of microRNA‐130b in Placental PGC‐1α/TFAM Mitochondrial Biogenesis Pathway,” Biochemical and Biophysical Research Communications 487, no. 3 (2017): 607–612, 10.1016/j.bbrc.2017.04.099. 28433632 PMC5522006

[37] T. I. Tamimi , H. M. Elgouhari , N. Alkhouri , et al., “An Apoptosis Panel for Nonalcoholic Steatohepatitis Diagnosis,” Journal of Hepatology 54, no. 6 (2011): 1224–1229, 10.1016/j.jhep.2010.08.023. 21145805 PMC3098936

[38] G. Kim , C. Giannini , B. Pierpont , et al., “Longitudinal Effects of MRI‐Measured Hepatic Steatosis on Biomarkers of Glucose Homeostasis and Hepatic Apoptosis in Obese Youth,” Diabetes Care 36, no. 1 (2013): 130–136, 10.2337/dc12-0277. 22933439 PMC3526202

[39] C. Mandelia , E. Collyer , S. Mansoor , et al., “Plasma Cytokeratin‐18 Level as a Novel Biomarker for Liver Fibrosis in Children With Nonalcoholic Fatty Liver Disease,” Journal of Pediatric Gastroenterology and Nutrition 63, no. 2 (2016): 181–187, 10.1097/MPG.0000000000001136. 26835904

[40] V. Nobili , A. Mantovani , S. Cianfarani , et al., “Prevalence of Prediabetes and Diabetes in Children and Adolescents With Biopsy‐Proven Non‐Alcoholic Fatty Liver Disease,” Journal of Hepatology 71, no. 4 (2019): 802–810, 10.1016/j.jhep.2019.06.023. 31279904

[41] F. Liu , J.‐M. Zhao , H.‐Y. Rao , et al., “Second Harmonic Generation Reveals Subtle Fibrosis Differences in Adult and Pediatric Nonalcoholic Fatty Liver Disease,” American Journal of Clinical Pathology 148, no. 6 (2017): 502–512, 10.1093/ajcp/aqx104. 29165568

[42] C. C. Cohen , E. Castillo‐Leon , A. B. Farris , et al., “PRO‐C3, a Serological Marker of Fibrosis, During Childhood and Correlations With Fibrosis in Pediatric NAFLD,” Hepatology Communications 5, no. 11 (2021): 1860–1872, 10.1002/hep4.1766. 34558828 PMC8557318

[43] A. Prats‐Puig , F. Ortega , J. Mercader , et al., “Changes in Circulating microRNAs Are Associated With Childhood Obesity,” Journal of Clinical Endocrinology and Metabolism 98, no. 10 (2013): E1655–E1660, 10.1210/jc.2013-1496. 23928666

[44] R. Shah , V. Murthy , M. Pacold , et al., “Extracellular RNAs Are Associated With Insulin Resistance and Metabolic Phenotypes,” Diabetes Care 40, no. 4 (2017): 546–553, 10.2337/dc16-1354. 28183786 PMC5360281

[45] A. Braza‐Boïls , J. Marí‐Alexandre , P. Molina , et al., “Deregulated Hepatic microRNAs Underlie the Association Between Non‐Alcoholic Fatty Liver Disease and Coronary Artery Disease,” Liver International 36, no. 8 (2016): 1221–1229, 10.1111/liv.13097. 26901384

[46] K. Mukherjee , B. Ghoshal , S. Ghosh , et al., “Reversible HuR‐microRNA Binding Controls Extracellular Export of miR‐122 and Augments Stress Response,” EMBO Reports 17, no. 8 (2016): 1184–1203, 10.15252/embr.201541930. 27402548 PMC4967961

[47] T. Iguchi , N. Niino , S. Tamai , K. Sakurai , and K. Mori , “Comprehensive Analysis of Circulating microRNA Specific to the Liver, Heart, and Skeletal Muscle of Cynomolgus Monkeys,” International Journal of Toxicology 36, no. 3 (2017): 220–228, 10.1177/1091581817704975. 28460582

[48] X. Liu , S. Chen , and L. Zhang , “Downregulated microRNA‐130b‐5p Prevents Lipid Accumulation and Insulin Resistance in a Murine Model of Nonalcoholic Fatty Liver Disease,” American Journal of Physiology. Endocrinology and Metabolism 319, no. 1 (2020): E34–E42, 10.1152/ajpendo.00528.2019. 32228319

[49] E. Isokuortti , Y. Zhou , M. Peltonen , et al., “Use of HOMA‐IR to Diagnose Non‐Alcoholic Fatty Liver Disease: A Population‐Based and Inter‐Laboratory Study,” Diabetologia 60, no. 10 (2017): 1873–1882, 10.1007/s00125-017-4340-1. 28660493

[50] J. A. Africa , C. A. Behling , E. M. Brunt , et al., “In Children With Nonalcoholic Fatty Liver Disease, Zone 1 Steatosis Is Associated With Advanced Fibrosis,” Clinical Gastroenterology and Hepatology 16, no. 3 (2018): 438–446.e1, 10.1016/j.cgh.2017.02.030. 28286193 PMC5589478

[51] E. M. Brunt , D. E. Kleiner , L. A. Wilson , et al., “Portal Chronic Inflammation in Nonalcoholic Fatty Liver Disease (NAFLD): A Histologic Marker of Advanced NAFLD. Clinicopathologic Correlations From the Nonalcoholic Steatohepatitis Clinical Research Network,” Hepatology 49, no. 3 (2009): 809–820, 10.1002/hep.22724. 19142989 PMC2928479

[52] J. B. Schwimmer , W. Dunn , G. J. Norman , et al., “SAFETY Study: Alanine Aminotransferase Cutoff Values Are Set Too High for Reliable Detection of Pediatric Chronic Liver Disease,” Gastroenterology 138, no. 4 (2010): 1357–1364, 1364.e1‐2., 10.1053/j.gastro.2009.12.052. 20064512 PMC2846968

[53] J.‐M. Schwarz , S. M. Noworolski , A. Erkin‐Cakmak , et al., “Effects of Dietary Fructose Restriction on Liver Fat, De Novo Lipogenesis, and Insulin Kinetics in Children With Obesity,” Gastroenterology 153, no. 3 (2017): 743–752, 10.1053/j.gastro.2017.05.043. 28579536 PMC5813289

[54] J. B. Schwimmer , P. Ugalde‐Nicalo , J. A. Welsh , et al., “Effect of a Low Free Sugar Diet vs Usual Diet on Nonalcoholic Fatty Liver Disease in Adolescent Boys: A Randomized Clinical Trial,” Journal of the American Medical Association 321, no. 3 (2019): 256–265, 10.1001/jama.2018.20579. 30667502 PMC6440226

[55] A. Moran , D. R. Jacobs , J. Steinberger , et al., “Insulin Resistance During Puberty: Results From Clamp Studies in 357 Children,” Diabetes 48 (1999): 2039–2044. 10512371 10.2337/diabetes.48.10.2039

[56] M. I. Goran and B. A. Gower , “Longitudinal Study on Pubertal Insulin Resistance,” Diabetes 50 (2001): 2444–2450. 11679420 10.2337/diabetes.50.11.2444

